# Fc-Elabela mitigates heart failure without liver and renal toxicity in mice

**DOI:** 10.3389/fphar.2025.1555728

**Published:** 2025-07-14

**Authors:** Huifen Zhou, Da-Wei Gong, Ling Chen, Hui Chen, Quan Wang, Mustajab Ullah, Saleem Ahmad, Ishtiaq Jeelani, Qingbin Zhao

**Affiliations:** ^1^ Department of Pathology, Hubei University of Science and Technology, Xianning, Hubei, China; ^2^ Division of Endocrinology, Department of Medicine, University of Maryland School of Medicine, Baltimore, MD, United States; ^3^ Division of Physiology, Department of Medicine, University of Maryland School of Medicine, Baltimore, MD, United States; ^4^ Department of Pharmacy, Central Hospital of Xianning Hubei, Xianning, China; ^5^ Tongji Hospital Affiliated to Tongji Medical College, Huazhong University of Science and Technology, Wuhan, China; ^6^ Department of Cell Biology and Physiology, University of Kansas Medical Center, Kansas, KS, United States; ^7^ Division of Endocrinology and Metabolism, School of Medicine, University of California, Irvine, CA, United States; ^8^ Department of Geratology, The First Affiliated Hospital of Xi’an Jiaotong University, Xi’an, Shaanxi, China

**Keywords:** Elabela, Fc fusion protein, heart failure, vascular endothelial growth factor receptor, myocardial infarction

## Abstract

**Aims:**

Elabela (ELA) is a ligand of the APJ receptor and exhibits anti-heart failure activities. However, the short half-life of the ELA limits its clinical applications. Our previous study recombined the short peptide ELA-21 and the Fc fragment of human IgG into a long-acting Fc-ELA-21 fusion protein and has shown that Fc-ELA-21′half-life in mice is 44 h and retained activation of the APJ receptor to exert anti-heart failure activity However, the anti-heart failure mechanisms of Fc-ELA-21 are still unclear, and its optimal dose range and long-term in vivo safety profile remain undefinedThis study aimed to investigate the anti-heart failure mechanisms of Fc-ELA-21, dose range, and *in vivo* safety.

**Methods and results:**

We investigated the effects of different doses of Fc-ELA-21 on cardiac function and potential signaling pathways and liver and kidney function by subcutaneous administration of Fc-ELA-21 in mice with myocardial infarction (MI)over 4 weeks. We found that Fc-ELA-21 significantly improved cardiac systolic dysfunction, mitigated pulmonary congestion, slowed down weight gain, activated vascular endothelial growth factor receptor 3 (VEGFR3) and APJ-mediated extracellular signal-regulated kinase (ERK) 1/2 signaling, and promoted endothelial cell proliferation in post-infarct mice. Moreover, the structure and function of the liver and kidney were normal in Fc-ELA-21-treated mice.

**Conclusion:**

Our results demonstrate that Fc-ELA-21 improves systolic heart failure by activating VEGFR3 signaling and suggest a mechanism for cross-talk between the APJ receptor and VEGFR3 in myocardial infarction MI. Moreover, Fc-ELA-21 is safe *in vivo*. Hence, the administration of Fc-ELA-21 fusion protein could be a novel therapeutic for systolic heart failure.

## 1 Introduction

Heart failure is the end-stage manifestation of cardiovascular diseases. Although there are various therapeutics for heart failure, the overall mortality from heart failure remains high (Jaarsma et al., 2018). Therefore, the development of new and more effective anti-heart failure therapeutics has been a research hotspot in this field. APJ, a G protein-coupled receptor, is widely expressed in human tissues and organs such as the central nervous system, heart, lung, kidney, adipose tissue, and muscles (O'Dowd et al., 1993). Studies have confirmed that APJ receptors play an important role in the physiological and pathological processes of the cardiovascular system and participate in a variety of cardiovascular diseases, such as atherosclerosis, coronary heart disease, pulmonary hypertension (PAH), hypertension, myocardial ischemia and reperfusion injury (Huang et al., 2019)[3]. APJ has long been considered an orphan receptor and it was not until 1998 that Apelin was found to be an endogenous ligand (Tatemoto et al., 1998). The Apelin/APJ signaling pathway regulates the physiological and pathological effects of the cardiovascular system (Hu et al., 2016; Xie et al., 2017), and Apelin activated APJ has a strong positive inotropic effect on the heart (Bertrand et al., 2018).

Recent studies have found that Elabela (also known as Toddler or Apela, ELA) is a new endogenous ligand for the APJ receptor, but it has little similarity to Apelin’s sequence (Chng et al., 2013; Pauli et al., 2014). Human ELA is a peptide containing 54 amino acids, including a signal peptide and a peptide containing 32 amino acids (ELA-32). To date, some studies have reported interactions between the ELA and APJ in the body and downstream signaling pathways and concomitant physiological responses that are similar and distinct from those of the Apelin/APJ signaling. Treating APJ-overexpressing HEK293T cells with ELA can internalize APJ receptors, inhibit cAMP production, occur phosphorylation of extracellular signal-regulated kinase (ERK) 1/2 and weak intracellular calcium flux (Wang et al., 2015). ELA plays an important role in the development of the zebrafish embryonic heart (Paskaradevan and Scott, 2012; Huang et al., 2017; Lu et al., 2017). In addition, it was found that ELA activated APJ can reduce arterial pressure and have positive inotropic effect (Murza et al., 2016). In animal experiments, ELA/APJ can prevent stress overload-induced heart failure by inhibiting the expression of angiotensin-converting enzyme (ACE) and pathological angiotensin II signaling pathway (Sato et al., 2017). Another study reported that ELA can increase cardiac contractility and induce coronary artery dilation by activating ERK 1/2 signaling pathway in adult rats (Perjés et al., 2016). It was found that the cardioprotective effects of ELA act as a ligand for APJ receptors, and APJ knockout mice do not respond to treatment with ELA. Pregnant mice with ELA knockout showed preeclampsia, including hypertension and proteinuria, while exogenous ELA infusion significantly improved hypertension and proteinuria (Ho et al., 2017). ELA can also inhibit the activity of renin-angiotension system by downregulating the expression of FoxM1 and ACE, so as to play a cardioprotective role (Kuba et al., 2019). The expression of ELA was decreased in lung tissues of patients and rat models with PAH, and administration of exogenous ELA in PAH rats reduced right ventricular systolic pressure and mitigated right ventricular hypertrophy (Yang et al., 2017).

However, ELA as a short peptide has an intrinsic defect, that is, a short *in vivo* half-life. Thus, ELA can only be administrated continuously subcutaneously with a mini pump or intraperitoneally in previous studies, which is not suitable for clinical applications. Therefore, our research team first recombined the short peptide ELA-21 and the Fc fragment of human IgG into a long-acting Fc-ELA-21 fusion protein and demonstrated that Fc-ELA-21′half-life in mice was 44 h, and retained activation of the APJ receptor to exert anti-heart failure activity, while the short peptide ELA-21 had a half-life of only 13 min in mice (Xi et al., 2019).

At this time, the signaling mechanisms underlying the anti-heart failure of Fc-ELA-21 remain to be fully elucidated, and its dose range and *in vivo* safety are unknown. A recent study demonstrated that G protein–coupled receptors and vascular endothelial growth factor receptor 3 (VEGFR3) can potentially coordinate their signaling to regulate endothelial function (Ma et al., 2019). Coincidentally, the APJ receptor is also the G protein-coupled receptor. We hypothesized that the anti-heart failure effect of Fc- ELA-21 would be mediated by APJ receptor signaling and VEGFR3 signaling. Thus, the aim of this study was to investigate the novel anti-heart failure mechanisms of Fc-ELA-21, dose range, and *in vivo* safety, providing the evidence for its clinical transformation as a novel therapeutic for heart failure.

## 2 Methods

### 2.1 Synthesis of Fc-ELA-21 fusion protein

**FIGURE 1:**
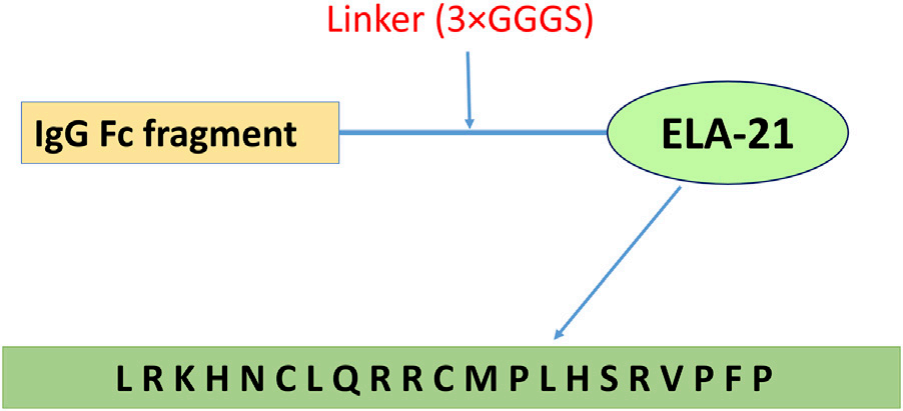
Fc-ELA-21 fusion protein design structure. Schematic representation of the Fc-ELA-21 construct, illustrating the N-terminal to C-terminal architecture: human IgG Fc fragment, a triple glycine-serine linker (3×GGGS), and the ELA-21 peptide (amino acid sequence: L-R-K-H-N-C-L-Q-R-R-C-M-P-L-H-S-R-V-P-F-P). This structure was previously developed in our laboratory (Xi et al., 2019) and serves as the long-acting therapeutic agent in this study.

The Fc-ELA-21 fusion protein was designed as shown in Figure 1 and synthesized by WuXi AppTec (Wuxi, China). The ELA-21 peptide was synthesized by GenScript (New Jersey, United States). Both were dissolved in autoclaved phosphate-buffered saline (PBS). For vehicle control experiments, PBS was added in volumes equivalent to those used for drug dilution in parallel experiments. For convenience, ELA-21 and Fc-ELA-21 were prepared at a concentration of 50 μL/10 g body weight for the targeted dose.

### 2.2 Animal studies

Animal studies were approved by the Institutional Animal Care and Use Committee of the University of Maryland School of Medicine. The investigation conformed to the Guide for the Care and Use of Laboratory Animals published by the US National Institutes of Health (NIH Publication No. 85-23, revised 1985). Male C57/BL6 wild-type mice (8 weeks old; Charles River Laboratories, Wilmington, MA, United States) were housed in a temperature- and light-controlled environment with free access to water. Myocardial infarction (MI) mouse models were established as follows: Mice were anesthetized via 2% isoflurane inhalation, and a pericardial incision was performed via thoracotomy. The left anterior descending coronary artery was permanently ligated at a site 2–3 mm from its origin using a 6–0 silk suture. Carprofen (5 mg kg^-1^) was administered as postoperative analgesia for 3 days.

MI mice were randomized into five groups (with 10 mice per group):

1. Vehicle/PBS (control),
2. ELA-21 (2 mg kg^-1^·d^-1^),
3. Fc-ELA-21 (4 mg kg^-1^·d^-1^),
4. Fc-ELA-21 (1 mg kg^-1^·d^-1^),
5. Fc-ELA-21 (0.1 mg kg^-1^·d^-1^).

Fc-ELA-21 and ELA-21 were administered subcutaneously at alternate sites daily for 4 weeks. At the end of the fourth week, MI mice were euthanized, and serum was immediately separated. The heart, lung, liver, and kidneys were harvested for further analysis.

### 2.3 Histology

The tissues of lung, liver, and kidney were fixed with 10% neutral buffered formalin (Sigma) and then were embedded in paraffin. 4 μm thick sections were prepared and stained with Hematoxylin and Eosin (HE). Three non-overlapping images were obtained per slide with a Olympus IX50 inverted microscope.

### 2.4 Echocardiography

Echocardiography in mice was described previously (Chen et al., 2010). Briefly, Cardiac function was evaluated by transthoracic echocardiography using Vevo 2,100 high-frequency, high-resolution ultrasound system with a 40-MHz linear transducer (MS-550 S; Visual Sonics, Toronto, Ontario, Canada) under 1.5% isoflurane inhalation anesthesia. Left ventricle morphology and systolic function were evaluated by two-dimensional M-mode recording.

### 2.5 Western blotting

Frozen heart and lung tissues were grinded and were dissolved and homogenized in ice-cold RIPA buffer (Thermo Fisher Scientific). Samples were then centrifuged at 12,000 *g* for 10 min at 4°C, and then the supernatant was collected. Protein concentrations were measured by the method of BCA (Thermo Fisher Scientific). Equal amounts (50 μg) of protein samples were loaded onto 10% SDS-PAGE and transferred to PVDF membranes (0.2 μm, Bio-Rad Laboratories). Protein levels were detected using Immobion Forte Western HRP Substrate (United States) with Image Lab system (United States). Quantification of the blots was measured by the Image Lab software (version 5.2.1, United States). The primary antibodies used were anti-phospho-ERK1/2 (1:1000, Cell Signaling), anti-ERK1/2 (1:1000, Cell Signaling), anti-proliferating cell nuclear antigen (PCNA) (1:1000, Cell Signaling), anti-VEGFR3 (1:1000, R&D Systems), purified ELA-21 immuno-rabbit serum (1:1000), and anti-β-actin (1:6000, R&D Systems). The second antibodies used were goat anti-rabbit IgG (1:5000, Invitrogen), goat anti-mouse IgG (1:5000, Jackon ImmunoResearch), and rabbit anti-goat IgG (1:5000, SeraCare).

### 2.6 Liver and kidney function

The levels of serum alanine aminotransferase (ALT) and aspartate transaminase (AST) were measured by using Reagent Kits (C164-0A, C154-0A, Catachem, United States) for evaluating liver function in MI mice treated with different drugs for 4 weeks according to the manufacturer’s instructions. The serum creatinine concentration in MI mice was measured by using QuantiChrom™ Creatinine Assay Kit (DICT-500, BioAssay Systems, United States) for evaluating kidney function according to the manufacturer’s instructions.

### 2.7 Data analysis

All data were presented as mean values ±SEM. Statistical significance between two experimental groups was determined using Student’s two-tailed *t*-test. Comparisons of parameters among more than three groups were analyzed by one-way ANOVA. When a comparison was done for groups with two factors, two-way ANOVA was used. A *P* value <0.05 was considered statistically significant. GraphPad Prism 7.00 Software (United States) was used for statistical analysis.

## 3 Results

### 3.1 Effective subcutaneous absorption of Fc-ELA-21 into circulation

**FIGURE 2:**
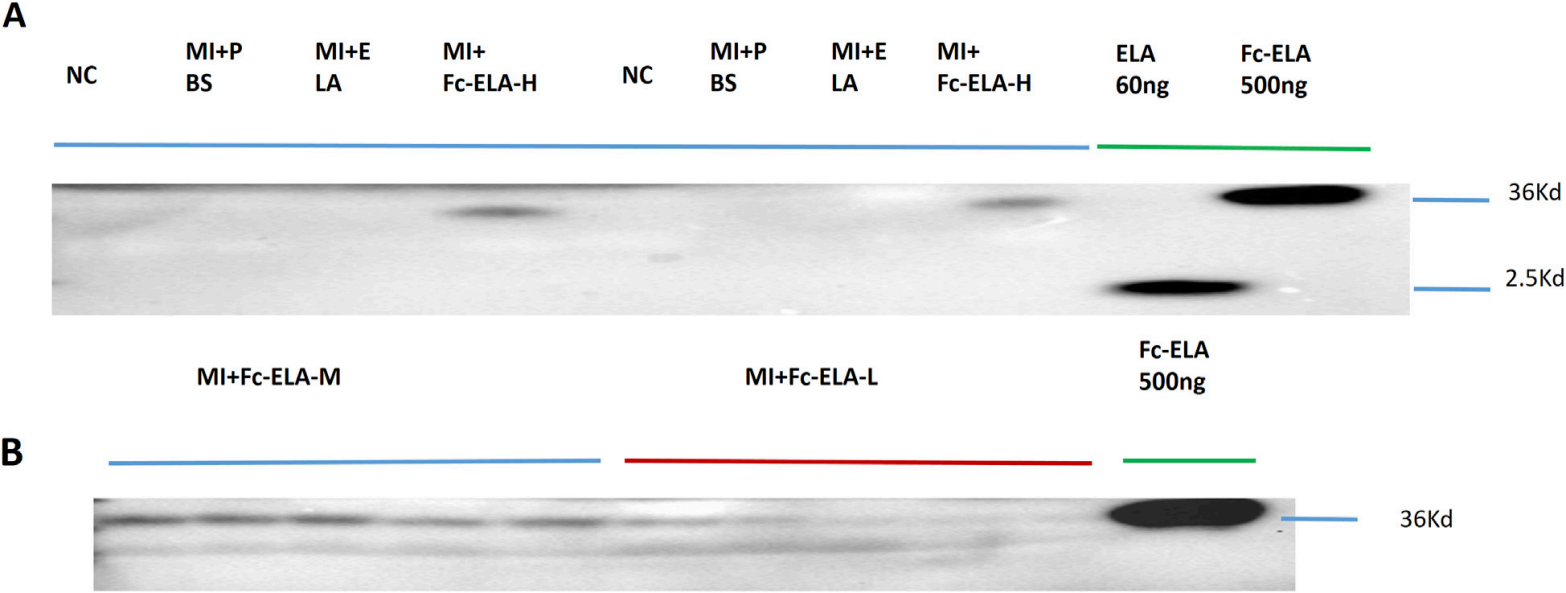
Fc-ELA-21 absorbed subcutaneously into blood circulation. **(A)** Representative Western blot analysis of serum samples from MI mice treated with high-dose Fc-ELA-21 (4 mg kg^-1^·d^-1^, Fc-ELA-H) for 4 weeks. Lanes include protein standards (500 ng Fc-ELA-21 and 60 ng ELA-21). The 36 kDa band corresponds to the intact Fc-ELA-21 fusion protein, while the 2.5 kDa band in the Fc-ELA-H lane is likely a degradation product or non-specific binding. **(B)** Western blot validation of Fc-ELA-21 absorption at medium (1 mg kg^-1^·d^-1^, Fc-ELA-M) and low (0.1 mg kg^-1^·d^-1^, Fc-ELA-L) doses. ELA-21 was undetectable in PBS control or ELA-21-treated groups, confirming the fusion protein’s prolonged systemic exposure.

We injected MI mice subcutaneously once daily for 4 weeks with different doses of Fc-ELA-21, and the serum was collected at the end of the fourth week. It was found that different doses of Fc-ELA-21 were able to be absorbed into circulation, whereas no ELA-21 was found in the blood circulation of MI mice administered with PBS or ELA-21 (Figure 2). These data support that the half-life of Fc-ELA-21 is significantly longer than that of ELA-21.

### 3.2 Fc-ELA-21 improves cardiac systolic dysfunction

**FIGURE 3:**
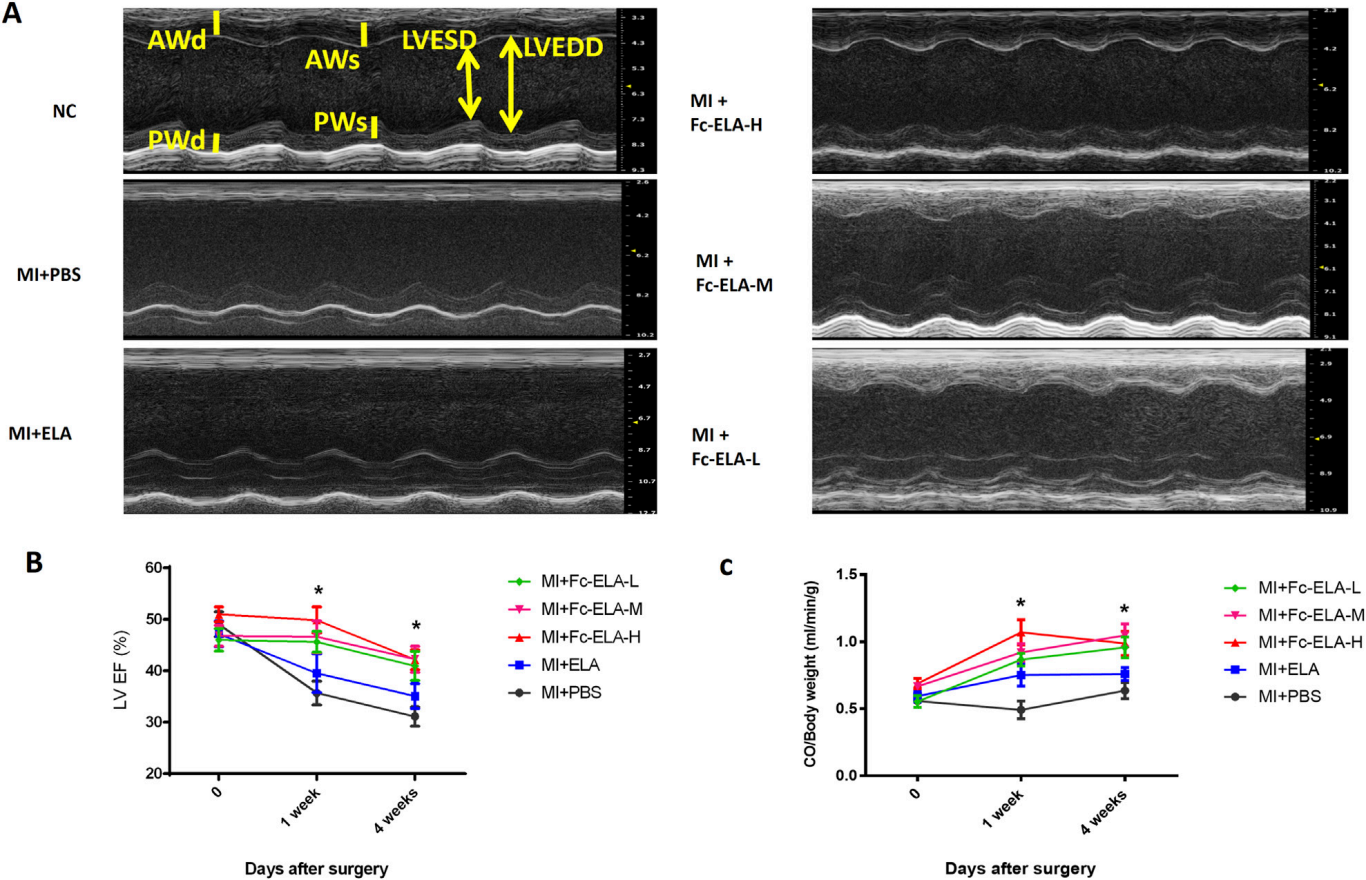
Fc-ELA-21 improves cardiac systolic dysfunction in MI mice. **(A)** Representative M-mode echocardiograms of the left ventricle (LV) in MI mice after 4 weeks of treatment: Top: MI + PBS group showing impaired LV systolic function (enlarged LV end-systolic dimension). Middle: MI + Fc-ELA-H group demonstrating improved LV remodeling with reduced cavity size. Bottom: MI + Fc-ELA-M and Fc-ELA-L groups showing intermediate improvements in LV dimensions. **(B)** Quantitative analysis of left ventricular ejection fraction (LVEF). Data are presented as mean ± SEM (n = 8). *p < 0.05 vs. PBS (one-way ANOVA with Tukey’s *post hoc* test). **(C)** Cardiac output (CO) normalized to body weight, showing dose-independent enhancement of systolic function by Fc-ELA-21.

The left ventricular ejection fraction (LVEF) and cardiac output (CO) per unit weight of MI mice treated with different doses of Fc-ELA-21 was significantly higher than that of mice treated with PBS or ELA-21. However, there was no significant dose response relationship between the different doses of Fc-ELA-21 (Figures 3A–C). These results demonstrate that Fc-ELA-21 improves cardiac systolic dysfunction.

### 3.3 Fc-ELA-21 mitigates pulmonary congestion

**FIGURE 4:**
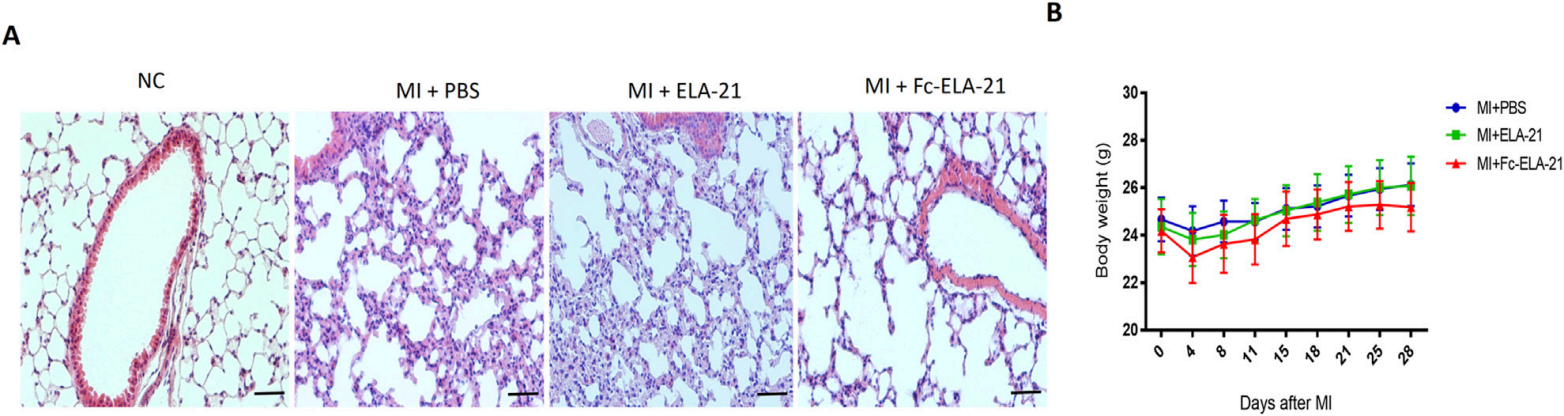
Fc-ELA-21 **(A)** Histopathological evaluation of lung tissues via HE staining. MI + PBS and MI + ELA-21 groups exhibit widened alveolar septa indicative of congestion, whereas MI + Fc-ELA-21 (4 mg kg^-1^·d^-1^) mice show normal alveolar architecture. Scale bar = 50 μm. **(B)** Longitudinal body weight measurements over 4 weeks. Fc-ELA-21-treated mice (4 mg kg^-1^·d^-1^) exhibit significantly slower weight gain, suggesting a potential diuretic or anti-edema effect. Data are mean ± SEM (n = 8–10).

MI mice treated with different medications were sacrificed at the end of the fourth week. HE staining of lung tissues showed that both PBS and ELA-21 treated mice had pulmonary congestion appearance, while high-dose Fc-ELA-21 treated mice had no significant pulmonary congestion (Fgure 4A–B), indicating that Fc-ELA-21 can mitigate pulmonary congestion. Moreover, we found that the weight of mice treated with high-dose Fc-ELA-21 increased more slowly than that of mice treated with PBS or ELA-21, suggesting that Fc-ELA-21 may have diuretic effect (Figure 4B).

### 3.4 Fc-ELA-21 activates ERK1/2 signaling

**FIGURE 5:**
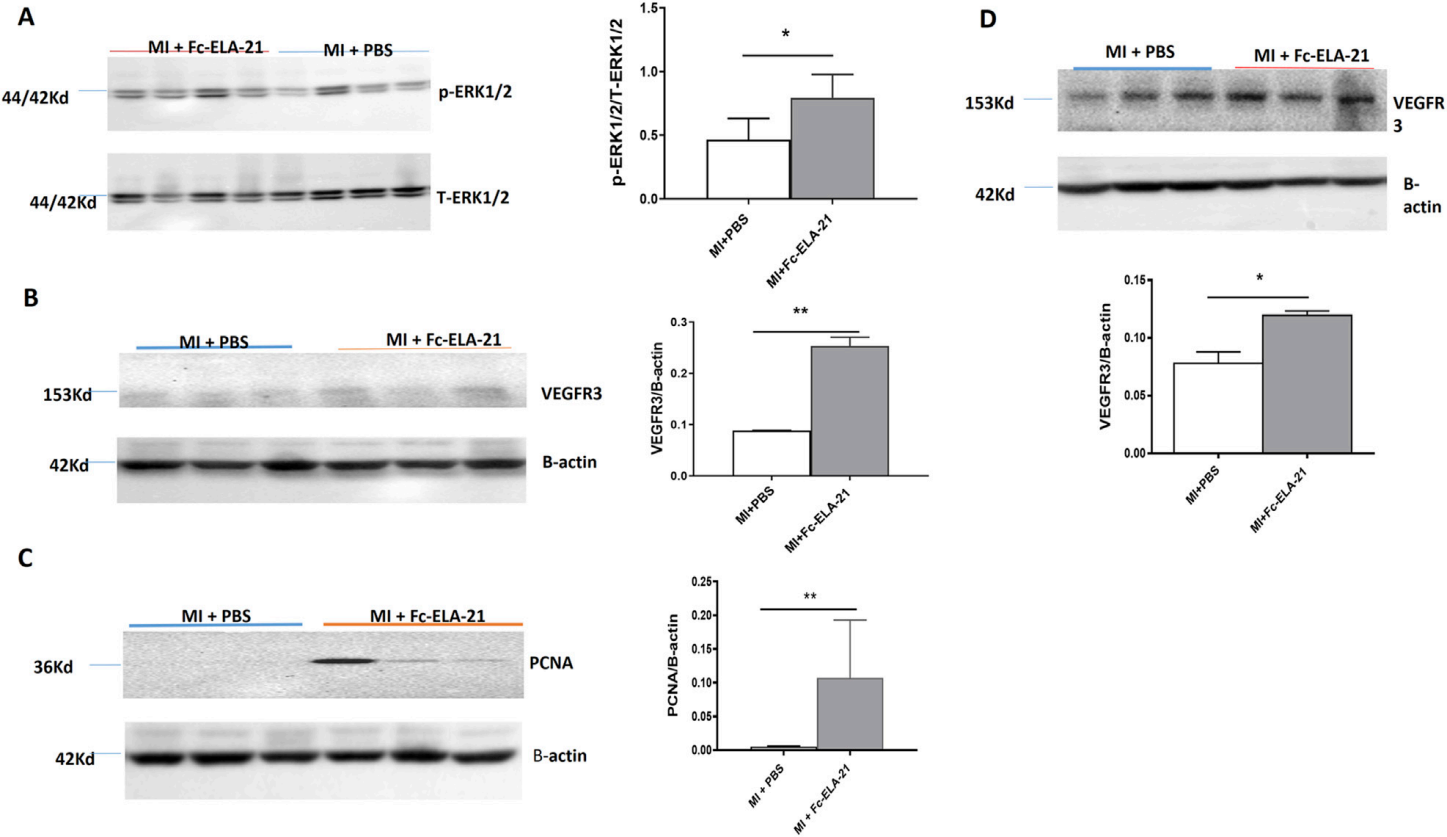
Fc-ELA-21 activates ERK 1/2 and VEGFR3 signalings and upregulates PCNA in MI mice. **(A–D)** Western blot analysis of myocardial and lung tissues: **(A)** Phosphorylated ERK1/2 (p-ERK1/2) expression in myocardium, indicating activation of the APJ-mediated ERK signaling pathway. **(B,C)** Upregulation of VEGFR3 and proliferating cell nuclear antigen (PCNA) in myocardium, suggesting promotion of endothelial cell proliferation and lymphangiogenesis. **(D)** Elevated VEGFR3 expression in lung tissue, reflecting systemic effects of Fc-ELA-21 on vascular endothelial function. Densitometric quantification of protein bands normalized to β-actin (n = 3–4). *p < 0.05, **p < 0.01 vs. PBS (Student’s t-test).

MI mice treated with different medications were sacrificed at the end of the fourth week. Western blot analysis showed that the expression of phosphorylated ERK 1/2 in the myocardium of high-dose Fc-ELA-21 treated mice was significantly higher than that of mice treated with PBS (Figures 5A–D), indicating that Fc-ELA-21 activates the ERK 1/2 signaling pathway in MI mice.

### 3.5 Fc-ELA-21 upregulates VEGFR3 and PCNA

Western blot analysis showed that the expression of VEGFR3 and PCNA in the myocardium of the high-dose Fc-ELA-21 treated mice was significantly higher than that in the PBS-treated mice, and VEGFR3 expression in the lung was also higher than that in PBS-treated mice (Figures 5A–D). These findings demonstrate that Fc-ELA-21 activates VEGFR3 signaling in MI mice which leads to endothelial cell proliferation, contributing to lymphangiogenesis or angiogenesis.

### 3.6 Effect of Fc-ELA-21 on liver and kidney functions

**FIGURE 6:**
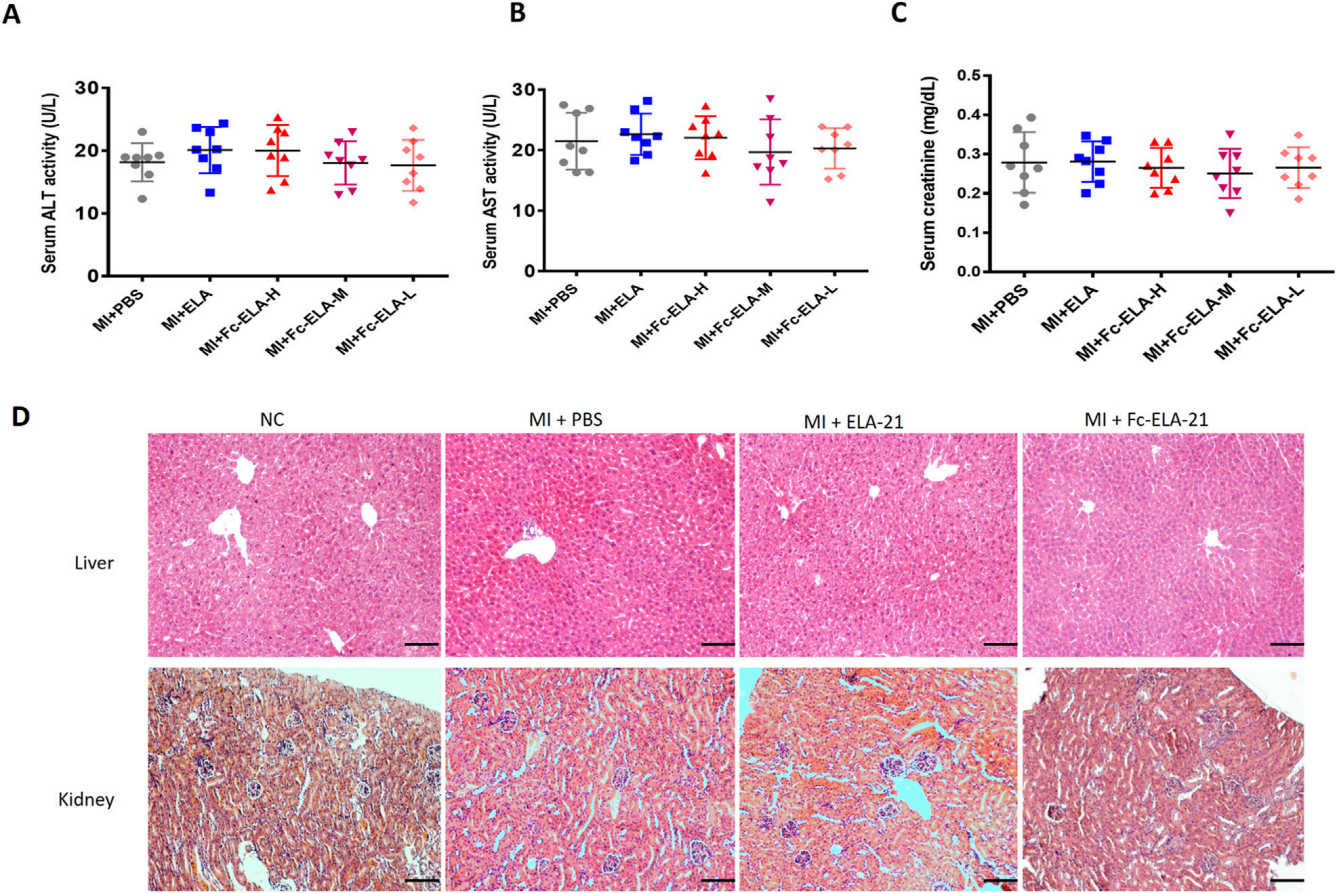
Effect of Fc-ELA-21 on the structure and function of liver and kidney in MI mice. **(A–C)** Serum biomarkers of hepatic (ALT, AST) and renal (creatinine) function, showing no significant differences between Fc-ELA-21-treated groups and PBS control (n = 8). **(D)** HE-stained sections of liver and kidney from MI + Fc-ELA-21 (4 mg kg^-1^·d^-1^) mice, displaying normal histological architecture without signs of inflammation or necrosis. Scale bar = 100 μm (n = 3).

We found that there was no significant changes in the levels of AST and ALT, and the creatine concentration in the serum of MI mice treated with different doses of Fc-ELA-21 compared with those of mice treated with PBS or ELA-21 at the end of the fourth week. Furthermore, no morphological abnormalities were observed in liver and kidney tissues (Figures 6A–D). These results suggest that the current Fc-ELA-21 dosage is safe *in vivo*.

## 4 Discussion

Our previous functional study of Fc-ELA-21 and ELA-21 used a dose of 0.3 mg kg^-1^. d^-1^ (Xi et al., 2019). In order to clarify the dose-response relationship of Fc-ELA-21, we subcutaneously injected MI mice using 4 mg kg^-1^. d^-1^, 1 mg kg^-1^. d^-1^, and 0.1 mg kg^-1^. d^-1^ of Fc-ELA-21, and used 2 mg kg^-1^. d^-1^ of ELA-21 as the control. In this study, we found that Fc-ELA-21 with a low dose of 0.1 mg kg^-1^. d^-1^ could also be absorbed into the circulation as those with a medium and high dose. The existence of Fc-ELA-21 in circulation was found by Western blotting technology, which laid a foundation for the fusion protein to play its role. However, ELA-21 was not found in the circulation of MI mice a few hours after administration, which further demonstrates that as a short peptide, it has an inherent defect, namely, a brief *in vivo* half-life, thus limiting its sustained anti-heart failure activity.

This study showed that Fc-ELA-21 at different doses could improve the left ventricular systolic dysfunction and cardiac output in MI mice, but there was no significant dose-response relationship. Thus, this study proposes for the first time that the lowest effective dose of Fc-ELA-21 is 0.1 mg kg^-1^. d^-1^. Simultaneously, we found that with the improvement of cardiac contractile function in Fc-ELA-21 treated MI mice, myocardial ERK 1/2 signaling was activated. A recent study demonstrated that Elabela binded to APJ receptors in the heart, increased cardiac contractility, and induced coronary vasodilation. The inotropic effect was accompanied by a significant increase in ERK 1/2 phosphorylation. Pharmacological inhibition of ERK 1/2 activation markedly attenuated the Elabela-induced inotropy (Perjés et al., 2016). Thus, this study demonstrates that Fc-ELA-21 can activate ERK 1/2 signaling like the short peptide ELA, thereby improving left ventricular systolic dysfunction in MI mice.

Importantly, we found a novel mechanism of Fc-ELA-21 against heart failure in this study, that is, Fc-ELA-21 activates VEGFR3 signaling leading to endothelial cell proliferation, which is beneficial to promotion of cardiopulmonary lymphangiogenesis or angiogenesis. VEGFRs belong to the family of receptor tyrosine kinases and play a central role in endothelial function, including cell proliferation and survival, angiogenesis, and lymphangiogenesis (Olsson et al., 2006). VEGFRs are activated by several closely related vascular endothelial growth factors (VEGFs) (Wirzenius et al., 2007; Coso et al., 2014), among which VEGF-C and VEGF-D preferentially recognize and activate VEGFR3, whose expression is mainly limited to lymphatic endothelial cells after embryonic development (Karkkainen et al., 2004; Milasan et al., 2019). VEGFR3 was also recently found to be expressed in the endothelial tip cells during angiogenesis and in tumor vasculature (Smith et al., 2010; Benedito et al., 2012). Lymphatic system regulates cardiac physiology and pathology, such as infammatory reactions (Kim and Song, 2017), tissue fluid balance (Breslin, 2014), reverse cholesterol transport (Martel et al., 2013) and atherosclerosis (Vuorio et al., 2014; Milasan et al., 2015) which can eventually influence heart function. In these studies, the obstruction of lymphatic flow led to subepicardial edema, depressed left ventricle (LV) contractile function, hemorrhages and arrhythmias (Cui, 2010). In addition, lymphangiogenic therapy with VEGFR3-specifc VEGF-C improved LV function in MI mice (Klotz et al., 2015). and enhanced cardiac edema and fibrosis in rats (Henri et al., 2016). VEGFR3 is the primary lymphangiogenic receptor for VEGF-C (Joukov et al., 1996). And VEGF-D (Achen et al., 1998) MI causes decreased cardiac lymph flow leading to edema both in humans (Nilsson et al., 2001) and in large animals (Ludwig et al., 1997). Cardiac edema can strongly regulate cardiac function and lead to dangerous arrhythmias [35] that are typically the main cause of sudden death post MI (Ludwig et al., 1997). In a recent clinical trial, the activation of both angiogenesis and lymphangiogenesis with adenoviral VEGF-D therapy was shown to be benefcial for myocardial perfusion and might have also improved cardiac fluid balance (Hartikainen et al., 2017). VEGFR3 plays an important role in cardiac lymphatic vessel morphology. The decreased VEGFR3 signaling makes mice face higher mortality, hemorrhage and structural changes of infarcted area, which suggests the importance of lymphatic vessel function in the healing post MIA recent study demonstrated that cardiac lymphatic system can influence the regenerative potential of the myocardium (Harrison et al., 2019). Furthermore, a recent study demonstrated that common therapeutic targets, such as G protein–coupled receptors and VEGFRs, can potentially coordinate their signaling through the adapter protein β-arrestin1 (ARRB1) to regulate endothelial function. In this study, we demonstrate that Fc-ELA-21 activates APJ receptor-mediated ERK 1/2 and VEGFR3 signalings in MI, suggesting a mechanism for cross talk between the APJ receptor and VEGFR3 in MI.

Moreover, this study found that Fc-ELA-21 attenuated pulmonary congestion in MI mice, which may be related to the improvement of cardiac contractile function leading to increased cardiac output. Secondly, Fc-ELA-21 activated the VEGFR3 signaling in MI mice which may promote the lymphangiogenesis. Previous studies indicated that ELA can balance body fluids (Santoso et al., 2015). APJ knockout mice exhibit abnormal fluid homeostasis (Murza et al., 2016). ELA affected fluid homeostasis by increasing diuresis (Deng et al., 2015). Interestingly, this study found that Fc-ELA-21 slowed down the weight gain of MI mice, indicating that it may produce diuresis. Furthermore, our results demonstrate that there is no toxic effect of Fc-ELA-21 on liver and kidney *in vivo*.

There are some limitations in this study. First of all, we found a novel mechanism of Fc-ELA-21’s anti-systolic heart failure, that is, it activated both APJ receptor and VEGFR3 signaling pathways, but we did not clarify the signal hub of the cross talk. Secondly, our current study on the anti-heart failure effect and mechanisms of Fc-ELA-21 is only carried out in small animals. As there are great differences between small animals and human beings, we need to further clarify the mechanisms of Fc-ELA-21’s anti-heart failure in large animals, as well as the effective dose range, administration interval, and *in vivo* safety, so as to promote its clinical transformation.

In summary, we have identified a novel role for Fc-ELA-21 in anti-heart failure through VEGFR3 signaling that explains in part the endothelial cell proliferation we found in MI mice. Moreover, these results demonstrate that there is a cross talk between VEGFR3 and APJ receptors. Importantly, Fc-ELA-21 is safe *in vivo*. Hence, Fc-ELA-21 fusion protein may be a novel therapeutic for systolic heart failure.

## Data Availability

The raw data supporting the conclusions of this article will be made available by the authors, without undue reservation.
