# Supplementary material for: Fc-Elabela mitigates heart failure without liver and renal toxicity in mice
Source: Front Pharmacol. 2025 Jul 14;16:1555728. doi: 10.3389/fphar.2025.1555728 (PMC12301219; doi:10.3389/fphar.2025.1555728)
Supplement: Supplementary file 1 [file Presentation1.pptx]

## Slide 1
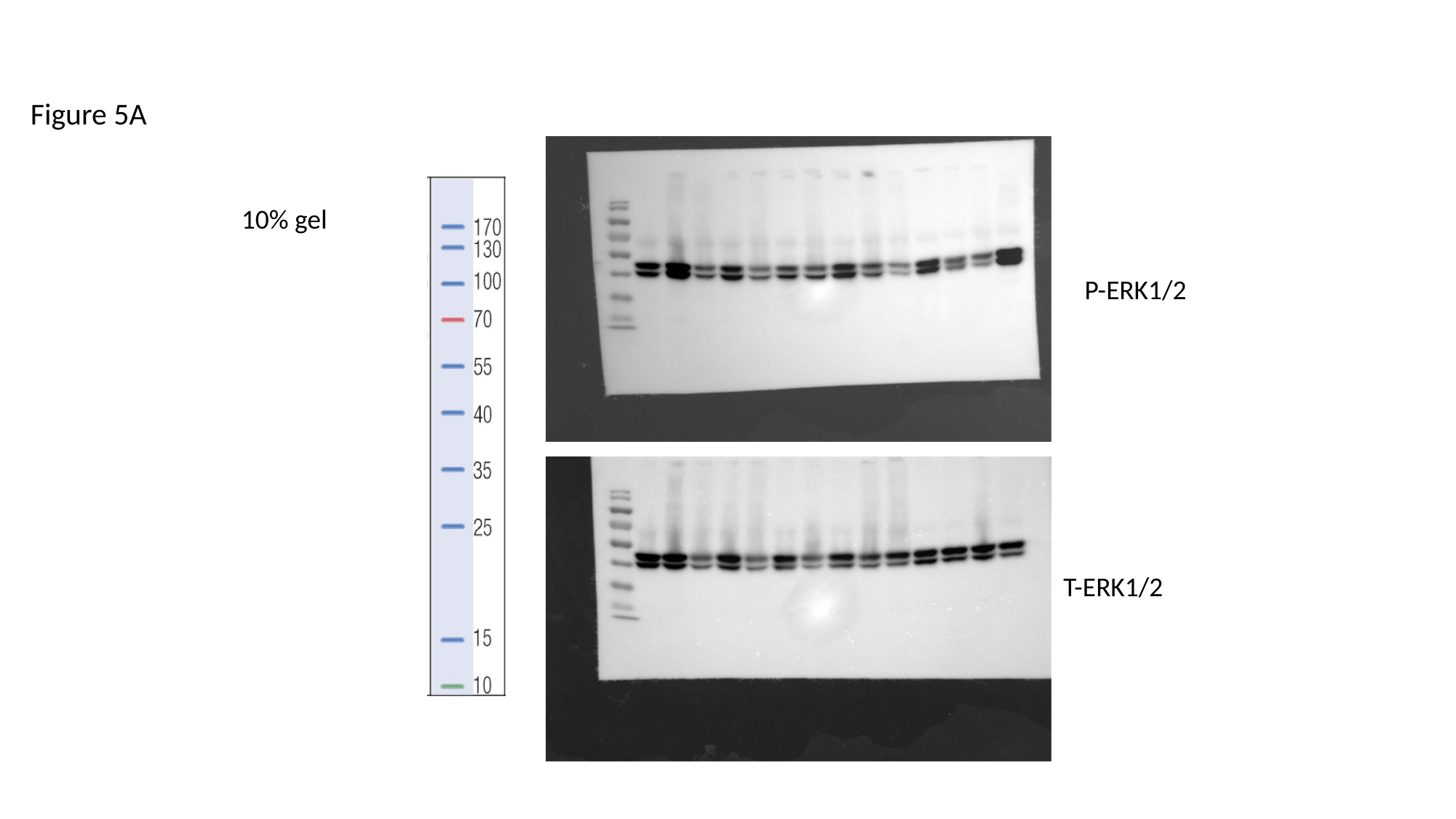

Figure 5A
10% gel
P-ERK1/2
T-ERK1/2

## Slide 2
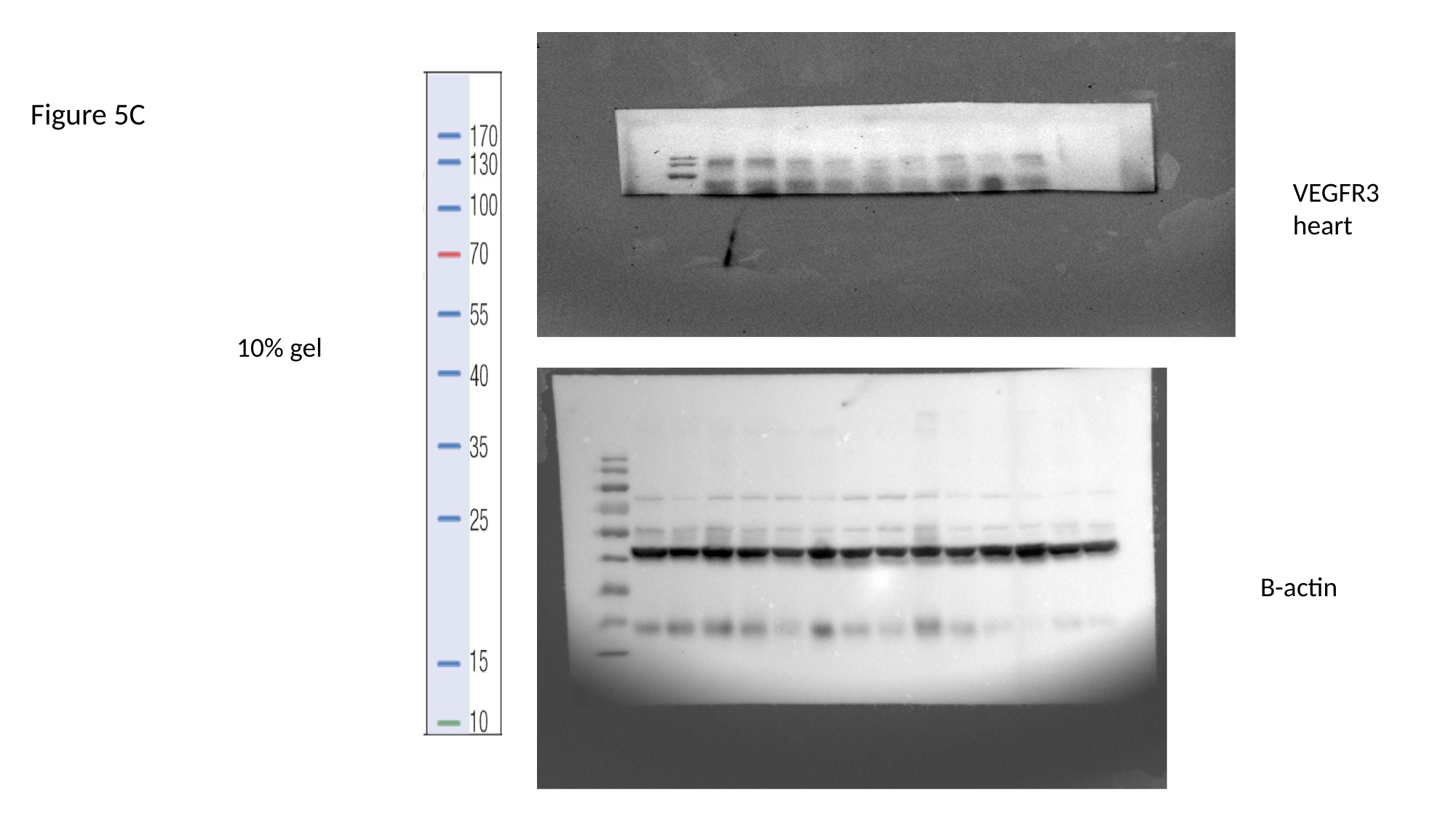

Figure 5C
VEGFR3
heart
10% gel
B-actin

## Slide 3
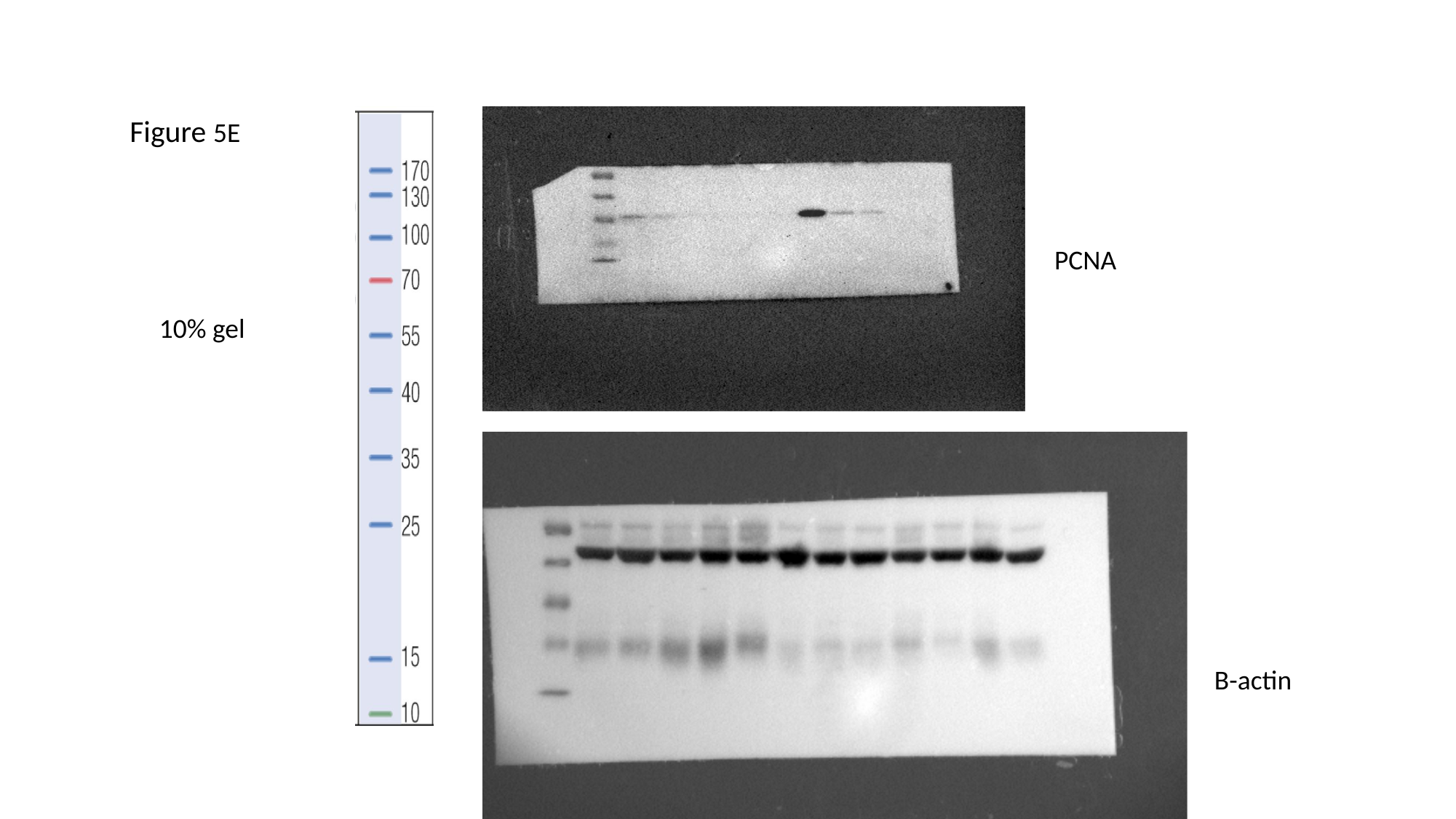

Figure 5E
PCNA
10% gel
B-actin

## Slide 4
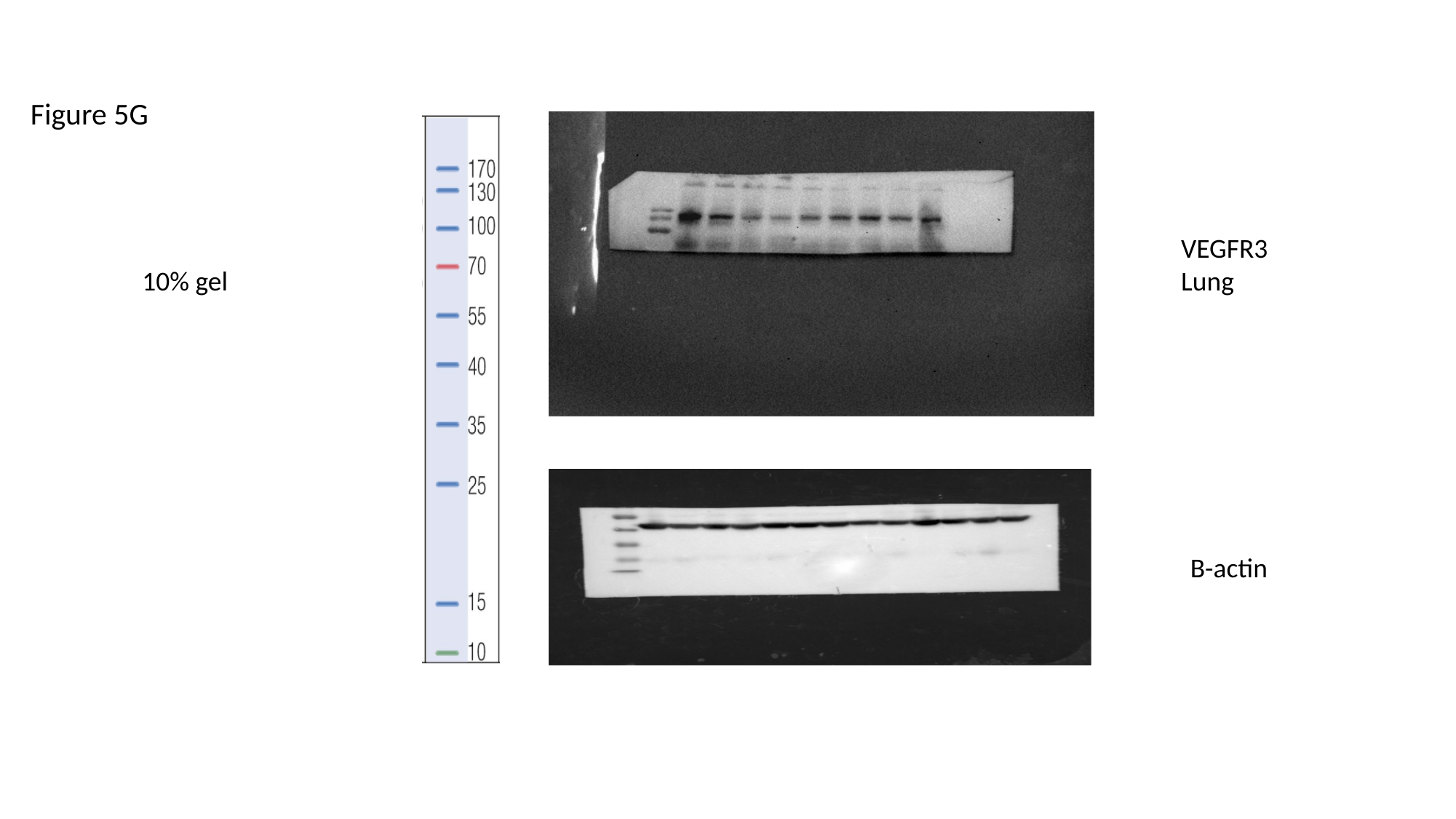

Figure 5G
VEGFR3
Lung
10% gel
B-actin
